# Associations between mental health competence and indicators of physical health and cognitive development in eleven year olds: findings from the UK Millennium Cohort Study

**DOI:** 10.1186/s12889-019-7789-7

**Published:** 2019-11-06

**Authors:** Steven Hope, Emeline Rougeaux, Jessica Deighton, Catherine Law, Anna Pearce

**Affiliations:** 1 Population, Policy and Practice Programme, UCL Great Ormond Street Institute of Child Health, 30 Guilford Street, London, WC1N 1EH UK; 2 Evidence Based Practice Unit, UCL and the Anna Freud Centre, 4-8 Rodney Street, London, N1 9JH UK

**Keywords:** Cohort studies, Positive mental health, Public health, Social and life-course epidemiology, children/child health, physical health, cognitive development

## Abstract

**Background:**

Positive mental health may support healthy development in childhood, although few studies have investigated this at a population level. We aimed to construct a measure of mental health competence (MHC), a skills-based assessment of positive mental health, using existing survey items in a representative sample of UK children, and to investigate its overlap with mental health difficulties (MHD), socio-demographic patterning, and relationships with physical health and cognitive development.

**Methods:**

We analysed the UK Millennium Cohort Study (MCS) when children were aged 11 years. Maternal (*n* = 12,082) and teacher (*n* = 6739) reports of prosocial behaviours (PS) and learning skills (LS) were entered into latent class models to create MHC measures. Using descriptive statistics, we examined relationships between MHC and MHD, and the socio-demographic patterning of MHC. Associations between MHC and physical health and cognitive development were examined with relative risk ratios [RRR] (from multinomial models): BMI status (healthy weight, overweight, obesity); unintentional injuries since age 7 (none, 1, 2+); asthma symptoms (none, 1, 2+); and tertiles of test scores for verbal ability, spatial working memory and risk-taking. Models were adjusted for potential confounding.

**Results:**

Four MHC classes were identified [percentages for maternal and teacher reports, respectively]: high MHC (high PS, high LS) [37%; 39%], high-moderate MHC (high PS, moderate LS) [36%; 26%]; moderate MHC (moderate PS, moderate LS) [19%; 19%]; low MHC (moderate PS, low LS) [8%; 16%]. Higher MHC was less common in socially disadvantaged children. While MHC and MHD were associated, there was sufficient separation to indicate that MHC captures more than the absence of MHD. Compared to children with high MHC, those in other MHC classes tended to have poorer physical health and cognitive development, particularly those with low MHC or high-moderate MHC. For example, children with maternal-report Low MHC were more likely to have experienced 2+ unintentional injuries (RRR: 1.5 [1.1–2.1]) and to have lower verbal ability scores (RRR: 2.5 [1.9–3.2]). Patterns of results were similar for maternal- and teacher-report MHC.

**Conclusion:**

MHC is not simply the inverse of MHD, and high MHC is associated with better physical health and cognitive development. Findings suggest that interventions to improve MHC may support healthy development, although they require replication.

## Background

The World Health Organization defines mental health as “a state of well-being in which every individual realizes his or her own potential, can cope with the normal stresses of life, can work productively and fruitfully, and is able to make a contribution to her or his community” [1]. It may promote health and life chances in childhood and adulthood [2, 3] and buffer against the effects of adversities, such as the negative impacts of socio-economic disadvantage on health and education outcomes [4]. At the level of evaluation trials, social and emotional learning programmes in schools have been demonstrated to improve aspects of mental health, such as social skills and self-regulation [5], and are associated with better educational and behavioural outcomes [6, 7]. However, there is limited evidence about childhood mental health at a population level. There are no agreed population measures of positive mental health for children [8, 9] and little research on its associations with indicators of physical health and cognitive development.

One conceptualisation of positive mental health is mental health competence (MHC) [8], which has been specifically developed for public health research, using a competence framework [10] to describe five developmental tasks and abilities children would be expected to achieve by early to mid-childhood: prosocial and helping behaviour, approaches to learning, overall social competence, responsibility and respect, and readiness to explore new things. Mental health competence [8, 11] is defined in relation to a dual continuum conceptualisation of mental health and difficulties, which proposes that positive mental health or competencies are not the inverse of mental health difficulties (MHD), and should be assessed in their own right [12, 13]*.* That is, even if they do not have a mental disorder and exhibit low levels of MHD, an individual will not necessarily experience high levels of positive mental health, or competencies; and associations with social determinants and health or developmental outcomes may differ for MHC and MHD. To date, MHC has only been described in Australian children at school entry age (using routine school census data), showing that levels of MHC are higher in girls and more advantaged groups [4, 11], and that MHC predicts learning skills [13].

We first sought to develop a population measure of MHC for a United Kingdom sample of children at age 11, which marks an important transition between childhood and adolescence, and between primary and secondary schooling. We used maternal and teacher reported data collected in the Millennium Cohort Study, the most recent nationally-representative UK cohort. As with the Australian work described above, our aim was to create a measure of MHC using data routinely collected in surveys and elsewhere for public health research. Second, we aimed to describe the relationship between MHC and MHD, in order to identify whether these measures are simply the inverse of one another, or whether a notable proportion of the cohort children could be identified as having high competence and high difficulties or low competence and low difficulties (as shown in Australia) [13]. Third, we investigated the social and demographic patterning of MHC with variables covering a range of social determinants of health. Finally, we aimed to extend the evidence base through examining associations between MHC and three prevalent indicators of child physical health (BMI status, unintentional injury and asthma symptoms) and three important aspects of cognitive development (verbal ability, spatial memory and risk-taking) in mid-childhood that could be plausibly influenced by differences in MHC. Specifically, we hypothesised that higher levels of MHC (indicating high prosocial and learning skills) would be associated with better physical health and cognitive development, after accounting for confounding.

## Methods

### Sample

We used the Millennium Cohort Study (MCS), a nationally-representative contemporary cohort of 19,517 children born in the UK between September 2000 and January 2002 [14]. We analysed data on children who were present at the age 11 sweep (13,112 singletons interviewed in 2012/13, 68% response rate). Only children living in England and Wales (*n* = 10,378) were eligible for inclusion in the teacher survey, and 7430 (72% response rate) participated [15]. After exclusion of those with missing data on mental health competence measures (described below), and where the main respondent was someone other than the mother, the analytic samples comprised 12,082 children for maternal report (92% of children present at that sweep) and 6739 children for teacher report (91% of children with data from the teacher survey). Multiple imputation was carried out for 20 imputed datasets under a missing at random assumption, to address missing outcome and covariate data, using Stata ‘mi impute chained’. Complete case analyses are included in the supplementary materials (Additional file 1: Tables S1-S4). Results are similar to those using the imputed data reported in the paper.

Ethical approval was granted by the Northern and Yorkshire multicentre research ethics committee in July 2011 (Ethics Committee reference: 11/YH/0203). Further information about the MCS can be found elsewhere (http://www.cls.ioe.ac.uk/MCS). Data were downloaded from the UK Data Service, University of Essex and University of Manchester, in March 2014.

### Measures

#### Mental health competence

We reviewed all questions included in the MCS 11-year sweep to determine those that would fall within previously reported, theory-based MHC domains [11]. We only selected questions that were positively-framed, reflecting the possession of attributes of the competencies assessed. We were able to identify questions relating to MHC prosocial behaviour and learning skills domains, reported by mothers and teachers. All of the maternal and most of the teacher questions were drawn from the Strengths and Difficulties Questionnaire (SDQ) [16]. Suitable questions were identified from three of the SDQ scales (hyperactivity [2 items], conduct problems [1 item], and prosocial behaviours [5 items]). Two additional questions on learning skills completed by the teacher were also included. Questions are included in Supplementary Materials (Additional file 1: Table S5).

#### Mental health difficulties

The SDQ Emotional symptoms scale, consisting of five items, was completed separately by mothers and teachers. Item responses were summed and dichotomised to identify the tertile of children with the greatest emotional difficulties [13]. Items from three other SDQ scales (Prosocial behaviour, Hyperactivity and Conduct problems) were included in the MHC exposure measure, and therefore these scales were not used as a measure of mental health difficulties.

### Outcomes

#### Physical health

Three indicators of physical health assessed in the MCS were included as outcomes. These are prevalent, plausibly linked to MHC, and have the potential to affect health outcomes throughout the lifecourse:

*Overweight and obesity:* children’s height and weight were measured by interviewers (using Tanita BF-522 W scales for weight and a Leicester stadiometer for height [17]). Body Mass Index (BMI; kg/m^2^) was categorised as healthy weight, overweight or obese using the International Obesity Task Force (IOTF) age and sex adjusted cut-offs for children [18].

*Injuries:* maternal report of the number of times the child was taken to the doctor, health centre, walk-in centre or hospital for an unintentional injury since the previous interview (for 91% of cohort members this was at age 7; an adjustment was made for those reporting injuries since an earlier interview). Injuries were categorised as none, one, and two or more.

*Asthma symptoms*: maternal report of whether the child had experienced serious symptoms of asthma, using items taken from the International Study of Asthma and Allergies in Childhood (ISAAC) core questionnaire for asthma (four or more wheeze attacks in the previous twelve months, one or more nights per week during which the child had their sleep disturbed by wheezing, or whether wheezing limited the child’s speech). Responses were summed and categorised as none (including children with controlled asthma), one, and two or more serious asthma symptoms.

#### Cognitive development

Results from three cognitive tests included in the MCS were used as outcomes, encompassing diverse aspects of cognitive development in mid-childhood:

*Verbal ability (verbal reasoning and verbal knowledge):* assessed using British Ability Scales (BAS) Verbal Similarities ability scores, accounting for the number and difficulty of test items and age of child at assessment [19].

Two of the Cambridge Neuropsychological Test Automated Battery (CANTAB) measures of neuropsychological development were also used in the MCS at age 11 [20].

*Spatial working memory:* tested the cohort child’s ability to retain spatial information and manipulate remembered items in working memory, assessed using measures of strategy, errors and response latencies. In this paper, we focus on the *strategy* outcome, reflecting the quality of the strategy used to complete each memory trial. Results for the other working memory measures were similar to those for strategy (not shown).

*Risk-taking:* the Cambridge Gambling Task assessed decision-making and risk-taking behaviour in a series of trials. Outcomes included quality of decision-making, deliberation time, delay aversion, overall proportional bet, risk adjustment and risk-taking. We present only the *risk-taking* outcome (results for the other risk-taking and decision-making outcomes replicate the findings presented, not shown).

Scores for all three markers of cognitive development were categorised into tertiles, reflecting the lowest, middle and highest thirds of the scoring distribution of each test. Verbal ability (mean = 59, range = 20–80) was grouped into low verbal skills (mean = 47, range = 20–54), medium skills (mean = 58, range = 55–61), and high verbal skills (mean = 69, range = 63–80). Spatial working memory (strategy) (mean = 34, range = 0–48) was grouped into poor strategy (mean = 27, range = 0–32), medium strategy (mean = 35, range = 33–36), and good strategy (mean = 39, range = 37–48). Risk-taking (mean = 0.53, range = 0.05–0.95) was grouped into low risk-taking (mean = 0.33, range = 0.05–0.45), medium risk-taking (mean = 0.53, range = 0.46–0.60), and high risk-taking (mean = 0.71, range = 0.61–0.95). Linear regression analyses of the continuous test scores by MHC class resulted in similar patterns to the results reported (not shown).

### Covariates

We investigated MHC in relation to a series of covariates in order to examine the socio-demographic patterning of MHC and to account for potential confounding between MHC and the outcomes: child sex and ethnicity; maternal age at cohort child’s birth and highest academic qualification; family structure (natural couple, reconstituted family, lone parent); and maternal mental health problems (Kessler-6 scale [21], summed and dichotomised using an existing cut-off for medium (including high) psychological distress [22]). We also investigated MHD as a potential confounder of the association between MHC and physical health and cognitive development in sensitivity analyses. This adjustment was not included in the main analyses due to uncertainties over the direction of the relationship between MHC and MHD (that is, if MHD is a mediator of the association between MHC and health and development, it would be an over-adjustment to account for it).

### Statistical analyses

#### Latent class analysis

Latent Class Analysis (LCA) was used to identify classes of MHC for maternal and teacher reports separately. Two sets of parameters were estimated for each latent class measure: ‘class membership probabilities’ (the prevalence of each class) and ‘item response probabilities’ (the combined probability of individuals in a given class displaying each of the MHC item responses) [23]. Models ranging from two to seven classes were considered (Supplementary Materials: Tables S6 and S7), with the following factors taken into account when selecting the final model: Akaike information criterion (AIC), Bayesian information criterion (BIC), class posterior probabilities (likelihood of members of an assigned class belonging to that class), and entropy (the precision of membership assignment across all individuals) [23]. The selection of the final classes was based on these measures of model fit and interpretability of the classes (that is, the extent to which each class was distinct, in terms of its MHC profile, from the others). Children were assigned to the class they had the highest probability of belonging to. 3% (*n =* 388) of children did not have information on all eight maternal-report items and 9% (*n* = 691) did not have information on all ten teacher-report items. Missing items were automatically imputed during the LCA procedure under a “missing at random” (MAR) assumption [24].

#### Associations between mental health competence, mental health difficulties, covariates and outcomes

We calculated weighted percentages for associations between MHC and MHD, and MHC and covariates. Multinomial regression was used to estimate relative risk ratios (RRRs) for outcomes, according to MHC classes (with 95% confidence intervals (CIs)), before and after adjustment for covariates.

Analyses were carried out in Stata/SE 15.1 (StataCorp LP, Texas, USA), including a Stata plug-in [20] for the SAS procedure PROC LCA [18]. All analyses were weighted to account for sample design and attrition at age 11.

## Results

### Latent measures of mental health competence

Four class latent measures were selected for both maternal and teacher reported MHC. The characteristics of MHC classes are detailed in Table 1 and described below.

**Table 1:** Latent class analysis probability estimates of classes of mental health competence (MHC) at age 11 in the UK Millennium Cohort Study

	Maternal-report (*n* = 12,082)				Teacher-report (*n* = 6739)			
	High MHC (Hi PS; Hi LS)	High-Moderate MHC (Hi PS; Mod LS)	Moderate MHC (Mod PS; Mod LS)	Low MHC (Mod PS; Low LS)	High MHC (Hi PS; Hi LS)	High-Moderate MHC (Hi PS; Mod LS)	Moderate MHC (Mod PS; Mod LS)	Low MHC (Mod PS; Low LS)
Low mental health competence response								
“Considerate” - *not true* (PS)	0.022	0.018	0.012	0.201	0.001	0.003	0.008	0.191
“Shares” - *not true* (PS)	0.017	0.011	0.026	0.196	0.010	0.014	0.018	0.145
“Helpful” - *not true* (PS)	0.010	0.005	0.024	0.121	0.010	0.011	0.016	0.172
“Kind” - *not true* (PS)	0.005	0.002	0.013	0.073	0.003	0.004	0.005	0.079
“Volunteers” - *not true* (PS)	0.006	0.011	0.050	0.203	0.011	0.031	0.125	0.395
“Thinks” - *not true* (LS)	0.006	0.112	0.090	0.795	0.007	0.047	0.035	0.532
“Tasks” - *not true* (LS)	0.017	0.129	0.125	0.644	0.011	0.101	0.029	0.599
“Obedient” - *not true* (LS)	0.016	0.022	0.034	0.303	0.005	0.012	0.018	0.102
“Independent” - *not well at all* (LS)	–	–	–	–	0.000	0.014	0.002	0.113
“Tries best” – *never* (LS)	–	–	–	–	0.000	0.001	0.001	0.029
Moderate mental health competence response								
“Considerate” - *somewhat true* (PS)	0.038	0.123	0.557	0.642	0.047	0.226	0.745	0.762
“Shares” - *somewhat true* (PS)	0.060	0.130	0.519	0.562	0.051	0.140	0.567	0.704
“Helpful”- *somewhat true* (PS)	0.031	0.027	0.482	0.474	0.069	0.099	0.830	0.658
“Kind” - *somewhat true* (PS)	0.014	0.023	0.333	0.394	0.032	0.040	0.589	0.607
“Volunteers” - *somewhat true* (PS)	0.110	0.298	0.636	0.536	0.228	0.365	0.726	0.441
“Thinks” - *somewhat true* (LS)	0.302	0.846	0.789	0.153	0.148	0.729	0.789	0.455
“Tasks” - *somewhat true* (LS)	0.225	0.687	0.647	0.300	0.036	0.745	0.593	0.384
“Obedient” - *somewhat true* (LS)	0.105	0.398	0.649	0.607	0.001	0.107	0.253	0.690
“Independent” - *not very well* (LS)	–	–	–	–	0.000	0.127	0.022	0.445
“Tries best” – *sometimes* (LS)	–	–	–	–	0.000	0.101	0.062	0.663
High mental health competence response								
“Considerate” - *certainly true* (PS)	0.939	0.859	0.430	0.157	0.953	0.770	0.247	0.047
“Shares” - *certainly true* (PS)	0.923	0.859	0.455	0.242	0.939	0.845	0.415	0.150
“Helpful” - *certainly true* (PS)	0.959	0.968	0.494	0.405	0.922	0.890	0.154	0.170
“Kind” - *certainly true* (PS)	0.981	0.975	0.653	0.533	0.965	0.956	0.406	0.314
“Volunteers” - *certainly True* (PS)	0.884	0.691	0.314	0.261	0.761	0.604	0.149	0.164
“Thinks” - *certainly true* (LS)	0.691	0.042	0.121	0.052	0.845	0.224	0.176	0.013
“Tasks” - *certainly true* (LS)	0.758	0.183	0.228	0.057	0.953	0.154	0.378	0.017
“Obedient” - *very true* (LS)	0.878	0.580	0.318	0.090	0.994	0.880	0.729	0.209
“Independently” - *usually* (LS)	–	–	–	–	0.116	0.648	0.541	0.414
“Tries best” - *usually* (LS)	–	–	–	–	0.162	0.632	0.720	0.291
“Independent” - *very well* (LS)	–	–	–	–	0.883	0.211	0.435	0.027
“Tries best” - *always* (LS)	–	–	–	–	0.837	0.265	0.217	0.017
*Prevalence (weighted)*	*37%*	*36%*	*19%*	*8%*	*39%*	*26%*	*19%*	*16%*

*MHC* mental health competence, *Hi PS* high prosocial behaviour, *Mod PS* moderate prosocial behaviour, *Hi LS* high learning skills, *Mod LS* moderate learning skills, *Low LS* low learning skills

#### Maternal-reported mental health competence

The largest class (high MHC: high prosocial behaviour, high learning skills), comprising 37% of children, displayed high levels of both prosocial behaviour and learning skills. This group was used as the baseline in regression analyses. The second largest class (high-moderate MHC: high prosocial behaviour, moderate learning skills), with 36% of children, included those with high ratings for prosocial behaviour items but a moderate rating for learning skills. The third (moderate MHC: moderate prosocial behaviour, moderate learning skills) comprised 19% of children, rated high for kindness and moderate for remaining prosocial behaviours and for learning skills. The fourth and smallest class (low MHC: moderate prosocial behaviour, low learning skills) included 8% of children who received moderate ratings on the prosocial behaviour items and low ratings on the learning skills items. Thus, there was greater differentiation in learning skills between the classes (low, moderate, or high) than in prosocial behaviour, which was high or moderate in all classes.

#### Teacher-reported mental health competence

Teacher-reported classes, which included two learning skills items that had not been asked of mothers, were similar in profile to maternal-reported MHC, and therefore classes were assigned the same labels. However, compared to the maternal-reported classes, prevalence was lower for the teacher-reported high-moderate MHC class (26%) and higher for the low MHC class (16%).

### Descriptive analyses

#### Dual continuum of mental health competence and mental health difficulties

Table 2 shows the relationship between MHC and MHD (reported separately by mothers and teachers). A notable proportion of children rated as high in MHC were also rated as having MHD. For example, 21% of children with high maternal-report MHC were rated as having difficulties. In contrast, 38% of the low MHC children were not rated as having MHD. Comparable associations were found between teacher-report MHC and difficulties. Sensitivity analyses using cohort child self-report of emotional difficulties (items from the Pediatric Quality of Life Inventory emotional subscale) resulted in a similar association between mental health difficulties and mental health competence (not shown).

**Table 2:** Associations between reported mental health competence and mental health difficulties scale at age 11 in the UK Millennium Cohort Study, weighted column % (n)

		Maternal-report (*n* = 12,082)				Teacher-report (*n* = 6739)			
		High MHC (Hi PS; Hi LS)	High-Moderate MHC (Hi PS; Mod LS)	Moderate MHC (Mod PS; Mod LS)	Low MHC (Mod PS; Low LS)	High MHC (Hi PS; Hi LS)	High-Moderate MHC (Hi PS; Mod LS)	Moderate MHC (Mod PS; Mod LS)	Low MHC (Mod PS; Low LS)
Mental health difficulties	No	79	67	65	38	76	58	66	43
	Yes	21	33	35	62	24	42	34	57

*MHC* mental health competence, *Hi PS* high prosocial behaviour, *Mod PS* moderate prosocial behaviour, *Hi LS* high learning skills, *Mod LS* moderate learning skills, *Low LS* low learning skills

#### Indicators of physical health and cognitive development

22% of children in the maternal-report sample were classified as overweight and 7% obese at age 11. 27% of children were reported to have had a single injury between ages 7–11, and 11% had two or more injuries. 3% of cohort children had one of the severe asthma symptoms and 1% had two or three symptoms. Tertiles of cognitive test scores were used in analyses based on the scoring distributions of each test in the MCS. Similar prevalences for the indicators were observed in the teacher-report sample.

#### Socio-demographic patterning of MHC

Girls, children in more advantaged socio-economic circumstances, and those whose mothers had not reported psychological distress were more likely to be classed as having high or high-moderate MHC (Table 3). The associations between ethnicity and MHC were more complex, potentially the consequence of small numbers of children from minority groups, particularly in the smaller, teacher-report sample.

**Table 3:** Characteristics of MHC classes in the UK Millennium Cohort Study, weighted row % (n)

		Maternal-report (*n* = 12,082)					Teacher-report (*n* = 6739)				
		All children Column %	High MHC (Hi PS; Hi LS)	High-Moderate MHC (Hi PS; Mod LS)	Moderate MHC (Mod PS; Mod LS)	Low MHC (Mod PS; Low LS)	All children Column %	High MHC (Hi PS; Hi LS)	High-Moderate MHC (Hi PS; Mod LS)	Moderate MHC (Mod PS; Mod LS)	Low MHC (Mod PS; Low LS)
Gender	Male	52	30	36	23	11	51	26	27	23	24
	Female	48	44	35	16	5	49	53	25	15	7
*Chi square test p-value*		*–*	*< 0.0001*				*–*	*< 0.0001*			
Ethnicity	White	86	36	37	19	8	86	39	27	19	15
	Mixed	3	32	36	23	9	3	38	20	21	21
	Indian	2	45	30	19	5	2	44	23	19	14
	Pakistani/Bangladeshi	4	46	28	19	8	4	37	22	25	17
	Black/ Black British	3	47	30	14	9	3	32	22	22	24
	Other Ethnic group	1	48	28	18	6	1	43	19	26	12
*Chi square test p-value*		*–*	*< 0.001*				*–*	*0.05*			
Maternal age at MCS birth (years)	14–19	9	27	41	20	13	8	26	25	23	26
	20–29	45	33	36	20	10	44	37	27	19	18
	30–39	40	42	35	18	5	42	44	25	20	11
	40+	6	38	32	21	9	7	36	29	17	18
*Chi square test p-value*		*–*	*< 0.0001*				*–*	*< 0.0001*			
Maternal academic qualification	Degree+	20	48	29	18	4	21	53	20	20	8
	Diploma	12	39	36	20	5	13	45	24	20	12
	A-levels	8	38	38	18	7	7	47	22	17	14
	GCSE grade A*-C	31	32	40	19	8	31	36	30	19	15
	GCSE D-G	11	29	37	21	13	12	29	28	22	22
	Other (including overseas)	7	35	34	20	11	6	29	22	22	27
	None	11	33	34	19	13	10	25	33	16	26
*Chi square test p-value*		*–*	*< 0.0001*				*–*	*< 0.0001*			
Family structure	Natural couple	62	43	35	18	5	63	44	25	19	12
	Reconstituted	12	25	40	21	14	12	32	28	19	22
	Lone	26	30	37	21	12	25	29	26	21	23
*Chi square test p-value*		–	*< 0.0001*				*–*	*< 0.0001*			
Maternal mental health	No-low distress	56	43	34	18	5	56	43	25	19	13
	Med/high distress	44	29	38	21	13	44	34	27	20	19
*Chi square test p-value*		*–*	*< 0.0001*				*–*	*< 0.0001*			
All children			37	36	19	8		39	26	19	16

*MHC* mental health competence, *Hi PS* high prosocial behaviour, *Mod PS* moderate prosocial behaviour, *Hi LS* high learning skills, *Mod LS* moderate learning skills; Low LS: low learning skills

### Association between mental health competence and indicators of physical health and cognitive development

Indicators of physical health and cognitive development varied by classes of MHC reported by mothers and teachers, with children rated as having higher MHC generally having better outcomes than those with lower MHC (Supplementary Materials: Additional file 1: Table S8). Multinomial regression was used to estimate the associations between MHC classes and indicators of health and cognitive development.

#### Overweight and obesity

For maternal-report MHC, the RRR for obesity was elevated for children classed as high-moderate MHC compared to the baseline class (high MHC), and this was only partly attenuated after adjustment for covariates (Table 4). There was no association between maternal-report MHC and overweight. For teacher-report MHC, risks of obesity were raised in all classes (compared to high MHC), and persisted after adjustment. As with maternal-report MHC, there was no association between teacher-report MHC and overweight.

**Table 4:** Associations between reported mental health competence and indicators of physical health and cognitive development at age 11 in the UK Millennium Cohort Study

		Maternal-report (*n* = 12,082)				Teacher-report (*n* = 6739)			
		High MHC (Hi PS; Hi LS)	High-Moderate MHC (Hi PS; Mod LS)	Moderate MHC (Mod PS; Mod LS)	Low MHC (Mod PS; Low LS)	High MHC (Hi PS; Hi LS)	High-Moderate MHC (Hi PS; Mod LS)	Moderate MHC (Mod PS; Mod LS)	Low MHC (Mod PS; Low LS)
Unadjusted Relative risk ratios (95% CI)									
Overweight	Healthy weight	–	–	–	–	–	–	–	–
	Overweight	–	1.1 (0.9–1.2)	0.8 (0.7–1.0)	1.1 (0.9–1.4)	–	1.2 (0.9–1.4)	1.1 (0.9–1.3)	1.0 (0.8–1.3)
	Obese	–	1.3 (1.1–1.6)	1.0 (0.8–1.3)	1.3 (0.9–1.9)	–	1.5 (1.1–2.0)	1.5 (1.1–2.1)	1.7 (1.2–2.4)
Injury	None	–	–	–	–		–	–	–
	1	–	1.2 (1.1–1.4)	1.0 (0.9–1.2)	1.2 (1.0–1.4)	–	1.2 (1.0–1.4)	1.0 (0.9–1.2)	1.2 (1.0–1.4)
	2+	–	1.7 (1.5–2.0)	1.5 (1.2–1.8)	1.8 (1.3–2.4)	–	1.3 (1.0–1.6)	1.2 (0.9–1.5)	2.1 (1.6–2.9)
Asthma symptoms	None	**–**	**–**	**–**	**–**	**–**	**–**	**–**	**–**
	1	**–**	1.1 (0.9–1.5)	1.0 (0.7–1.4)	1.1 (0.7–1.8)	–	1.2 (0.9–1.7)	0.8 (0.5–1.3)	0.8 (0.5–1.4)
	2+	**–**	1.0 (0.6–1.5)	0.8 (0.5–1.4)	2.0 (1.0–3.8)	–	1.2 (0.7–2.1)	0.7 (0.3–1.6)	2.2 (1.2–4.3)
BAS Verbal ability: tertiles	High verbal skills	–	–	–	–	–	–	–	–
	Medium verbal skills	–	1.3 (1.2–1.5)	1.3 (1.1–1.5)	1.7 (1.4–2.2)		1.3 (1.1–1.5)	1.1 (0.9–1.3)	1.6 (1.3–2.0)
	Low verbal skills	–	1.8 (1.5–2.0)	1.7 (1.5–2.0)	3.7 (2.8–4.7)	–	2.3 (1.9–2.7)	1.8 (1.5–2.2)	4.0 (3.2–5.0)
CANTAB Spatial working memory (strategy): tertiles	Good strategy	–	–	–	–	–	–	–	–
	Medium strategy	–	1.3 (1.1–1.5)	1.1 (0.9–1.3)	1.7 (1.3–2.3)	–	1.8 (1.5–2.1)	1.1 (0.9–1.4)	1.9 (1.5–2.4)
	Poor strategy	–	1.5 (1.3–1.8)	1.2 (1.0–1.5)	2.6 (2.1–3.3)	–	2.3 (1.9–2.8)	1.4 (1.1–1.6)	3.0 (2.5–3.7)
CANTAB risk-taking scores: tertiles	Low risk-taking	–	–	–	–	–	–	–	–
	Medium risk-taking	–	1.1 (0.9–1.2)	1.1 (0.9–1.3)	1.3 (1.0–1.7)	–	1.4 (1.2–1.7)	1.3 (1.1–1.6)	1.4 (1.1–1.7)
	High risk-taking	–	1.4 (1.2–1.6)	1.5 (1.3–1.8)	2.3 (1.8–3.0)	–	2.0 (1.7–2.3)	1.8 (1.5–2.2)	2.8 (2.2–3.6)
Adjusted Relative risk ratios* (95% CI)									
Overweight	Healthy weight	–	–	–	–	–	–	–	–
	Overweight	–	1.1 (0.9–1.2)	0.8 (0.7–1.0)	1.1 (0.8–1.4)	–	1.2 (1.0–1.4)	1.1 (0.9–1.3)	1.0 (0.8–1.3)
	Obese	–	1.2 (1.0–1.4)	0.9 (0.7–1.1)	1.0 (0.7–1.5)	–	1.3 (1.0–1.8)	1.5 (1.0–2.1)	1.5 (1.0–2.2)
Injury	None	–	–	–	–		–	–	–
	1	–	1.2 (1.0–1.3)	1.0 (0.9–1.1)	1.1 (0.9–1.3)	–	1.1 (0.9–1.3)	1.0 (0.9–1.2)	1.1 (0.9–1.4)
	2+	–	1.5 (1.3–1.8)	1.3 (1.1–1.7)	1.5 (1.1–2.1)	–	1.2 (1.0–1.6)	1.1 (0.9–1.5)	2.0 (1.5–2.8)
Asthma symptoms	None	–	–	–	–	–	–	–	–
	1	–	1.1 (0.8–1.4)	0.9 (0.7–1.3)	0.9 (0.6–1.5)	–	1.1 (0.8–1.6)	0.8 (0.5–1.2)	0.7 (0.4–1.2)
	2+	–	0.9 (0.6–1.3)	0.7 (0.4–1.2)	1.4 (0.8–2.7)	–	1.0 (0.6–1.7)	0.5 (0.2–1.3)	1.5 (0.7–3.0)
BAS Verbal ability: tertiles	High verbal skills	–	–	–	–	–	–	–	–
	Medium verbal skills	–	1.2 (1.1–1.4)	1.2 (1.0–1.4)	1.5 (1.2–1.9)		1.2 (1.0–1.4)	1.1 (0.9–1.3)	1.5 (1.2–1.9)
	Low verbal skills	–	1.5 (1.3–1.8)	1.6 (1.3–1.8)	2.5 (1.9–3.2)	–	2.0 (1.6–2.4)	1.8 (1.4–2.2)	3.3 (2.6–4.1)
CANTAB Spatial working memory (strategy): tertiles	Good strategy	–	–	–	–	–	–	–	–
	Medium strategy	–	1.2 (1.1–1.4)	1.0 (0.9–1.2)	1.5 (1.2–1.9)	–	1.7 (1.4–2.0)	1.1 (0.9–1.3)	1.6 (1.3–2.1)
	Poor strategy	–	1.4 (1.2–1.7)	1.1 (1.0–1.4)	2.1 (1.6–2.7)	–	2.1 (1.8–2.6)	1.3 (1.1–1.6)	2.5 (2.0–3.2)
CANTAB Risk-taking: tertiles	Low risk-taking	–	–	–	–	–	–	–	–
	Medium risk-taking	–	1.0 (0.9–1.2)	1.0 (0.8–1.2)	1.1 (0.8–1.5)	–	1.3 (1.1–1.5)	1.1 (0.9–1.3)	1.0 (0.8–1.3)
	High risk-taking	–	1.2 (1.1–1.4)	1.2 (1.0–1.5)	1.6 (1.2–2.2)	–	1.6 (1.3–1.9)	1.3 (1.0–1.6)	1.6 (1.3–2.1)

*MHC* mental health competence, *Hi PS* high prosocial behaviour, *Mod PS* moderate prosocial behaviour, *Hi LS* high learning skills, *Mod LS* moderate learning skills, *Low LS* low learning skills

*Adjusted for cohort member gender and ethnicity, maternal age at birth of cohort child, maternal academic attainment, maternal mental health and family structure

#### Injury

For maternal-report MHC, RRR of two or more injuries were raised for children in all classes compared to those with high MHC, and these were only partly attenuated after adjustment. Children identified as high-moderate or low MHC were also at a slightly increased risk of one unintentional injury, although this only remained the case for the high-moderate MHC class after adjustment. For teacher-report MHC, risks of experiencing two or more unintentional injuries were elevated for children with high-moderate MHC and, in particular, the low MHC class, before and after adjustment. Children in the high-moderate and low MHC classes were also at slightly greater risk of experiencing a single injury, however this was attenuated and non-significant after adjustment for covariates.

#### Asthma symptoms

For maternal-report MHC, RRR of having two or more severe asthma symptoms were elevated for children in the maternal-report low MHC class (compared to high MHC), and while this elevated risk was only partly attenuated after adjusting for covariates, it was no longer statistically significant. There was no association between maternal-report MHC and having single asthma symptoms. For teacher-report MHC, the same pattern of results was observed.

#### Cognitive development

For each of the three measures of cognitive development (verbal ability, spatial working memory and risk-taking), RRRs for scoring in the lowest tertile (indicating poorest performance) were elevated for children in all maternal-report MHC classes compared to those in the high MHC class, and this remained so after accounting for covariates. Risks of a low score were particularly raised for children in the low MHC class, and, in the working memory test, also for children in the high-moderate class. For teacher-report MHC, the same pattern was observed. Additionally, children in the high-moderate class were at particularly increased risk of scoring in the lowest tertile of the risk-taking test. Risks of scoring in the middle tertile were generally elevated in children who were in the low MHC and high-moderate MHC classes, although patterns varied by test and by whether MHC was reported by mothers or teachers.

#### Sensitivity analyses

Regression analyses were repeated for maternal-report MHC in a sample that was restricted to only those children with teacher-report MHC to examine the potential impact of selection bias in the teacher sample. The patterns of results were similar to those reported (not shown). In order to check that our findings were not an artefact of the four-class MHC measures chosen, we repeated the analyses using two class measures of MHC (capturing high and moderate-low MHC), which was deemed to be the second best measure in terms of model fit and interpretability. We also repeated analyses using an MHC score of summed responses to the individual MHC items. As with the four-class measure, children with lower MHC had generally poorer outcomes (results not shown). Regression analyses were repeated with the inclusion of the SDQ emotional problems scale as an additional covariate, in order to assess the association between MHC and outcomes after accounting for MHD. Despite some attenuation, the pattern of results was similar to those reported (Supplementary Materials: Additional file 1: Table S9).

## Discussion

### Summary of findings

In a nationally-representative contemporary sample of 11 year old UK children, we drew on existing survey items to develop a measure of mental health competence (MHC) for public health research comprising two age-relevant competencies: prosocial behaviours and learning skills, reported by mothers and teachers. Using a data-driven technique to summarise the combination and level of both competencies, four classes were identified ranging from high MHC (high prosocial behaviour and high learning skills) to low MHC (moderate prosocial behaviour and low learning skills). Although most children displayed high or high-moderate MHC, levels were lower in children from less advantaged socio-economic circumstances, boys, and children of mothers with psychological distress. MHC and mental health difficulties (MHD) were not the inverse of one another, with notable proportions of children rated with high MHC and high MHD, or low MHC and low MHD.

Using indicators of physical health and cognitive development that could be plausibly influenced by MHC, we showed variability in outcomes and degree of severity according to MHC class. MHC was not, or only weakly, associated with experiencing a single unintentional injury between ages 7–11 years, being overweight or having only a single symptom of asthma. MHC was more strongly related to injuries on multiple occasions, obesity and two or more asthma symptoms, although associations generally did not show a dose-response relationship according to level of MHC. For example, the likelihood of poor physical health on the indicators assessed was generally greater for those with low MHC (compared to those in the high MHC class); however, the elevated risks for those with moderate MHC were often smaller than those with high-moderate MHC. MHC was strongly associated with measures of cognitive development available in the MCS at 11 years (verbal, spatial working memory and risk-taking), with low MHC (and to a lesser extent high-moderate) associated with the lowest scores. In the MCS, as in many other studies, children from more disadvantaged circumstances are at greater risk of poorer physical health and cognitive development [19, 25]. While adjustment for confounding attenuated the associations between MHC and outcomes in some cases, elevated risks remained. Results were also similar after accounting for MHD as a covariate. However, given potential bidirectional pathways between mental health competence and difficulties, these sensitivity analyses represent a potential over-adjustment.

### Comparison with other findings

Positive mental health and mental illness do not necessarily represent opposite ends of a continuum of mental health, and, while related constructs, they may develop along separate pathways [26]. As with earlier research on MHC from Australia [13], we found that competence and mental health difficulties were distinct constructs, and that high MHC was not necessarily accompanied by low MHD (or low MHC with high MHD). Our findings are also consistent with earlier studies that have revealed social patterning in diverse measures of positive mental health [27–32], including mental health competence [11], whereby advantaged children are most likely to exhibit positive mental health. However, we have gone further by differentiating high, moderate and low functioning on prosocial behaviour and learning skills domains. We have shown that, although less prevalent in children from disadvantaged families, high MHC was common in MCS children at age 11 across all social groups.

There is limited research investigating associations between positive mental health and indicators of child health. For childhood overweight, evidence is inconsistent, with some research showing an association between higher levels of learning skills and prosocial behaviours and smaller risks of overweight and obesity [33–36], while others have found an association between higher levels of social skills and greater risks of overweight and obesity [37, 38]. Although there is evidence linking childhood problem behaviours with risk of injury [39], to our knowledge this is the first study to have demonstrated an association between positive mental health (specifically MHC) and injury. Asthma management has been associated in a number of studies with psychological factors (including coping styles and mental health problems) [40], with some evidence that specific positive mental health factors (such as self-efficacy) predict better asthma control [41]. In terms of cognitive development, Australian research has shown similar results to those observed in the MCS, with higher MHC at age 5 years associated with classroom oral communication skills [4], and language and cognitive skills [13]. Attitudes to risk may have an impact on important life decisions [42] and risky behaviours [43], and skills related to mental health competence are associated with a lower likelihood of initiating risk-taking behaviours in adolescence [44]. In this context, relationships between MHC and risk-taking scores at age 11 years may presage future patterns of behaviour.

Inconsistencies between studies may in part reflect non-linear relationships between positive mental health and outcomes. For example, our study has shown that children classed as moderate MHC sometimes had physical health and cognitive development outcomes as good as children with high MHC and better than children with high-moderate MHC. Patterns of association between MHC and outcomes are likely to, in part, reflect the greater differentiation manifest in learning skills (which could be classed into three levels) compared to prosocial behaviour (for which only high and moderate levels were identified).

### Strengths and limitations

This study was carried out using a large, nationally-representative cohort of children. We were able to investigate competencies, in contrast to measures of mental illness which seek to identify children with mental health difficulties versus a heterogeneous comparison group of children without difficulties. Using latent class analysis, we developed a measure of MHC which allowed questions on prosocial behaviours and learning skills to be summarised while differentiating levels of high and moderate functioning rather than producing a single score. Unlike other measures of positive mental health based on measuring subjective mood or affect, we focused on competencies than could be assessed by mothers and teachers. A limitation of the study is the relatively narrow range of questions pertinent to the measurement of MHC, which captured only learning skills and prosocial behaviours. As operationalised elsewhere [8], MHC (depending on the age of the child) might also include responsibility and respect, and readiness to explore new things. In the MCS, as in many population surveys that include assessments of health and well-being, negative markers of mental health predominate rather than strengths or competencies. Our review of relevant survey questions resulted in the selection of the majority of MHC items from the widely-used Strengths and Difficulties Questionnaire (SDQ), including all items from the prosocial scale and three positively-framed items from the hyperactivity and conduct disorders scales of the total difficulties scale. The fact that the items used to capture MHC in the present study are commonly collected in surveys and at different ages is, however, also a potential strength. We do not claim to have developed a complete measure of MHC, rather to have made efficient use of existing data to explore positive mental health at a population level, with a focus on the use of such measures in a public health and epidemiology setting. This provides opportunities for similar measures of MHC to be developed and used in other studies and at different stages of childhood and adolescence. There are challenges in deciding the most appropriate reporter for mental health-related measures as there tends to be relatively low agreement [45]. However, there is a general preference for taking more than one perspective, including in population studies [46]. In our study, the same classes of MHC were identified from reports provided by mothers and teachers, indicating a consistent structure for this measure of MHC, although prevalence of classes differed slightly. The patterning of results were largely consistent for mother and teacher reported MHC, although the strength of associations tended be greater for teacher-report MHC. Differences (or similarities) between mother and teacher-report MHC do not necessarily indicate bias, and may instead show the importance of context, with children’s behaviours varying between home and school settings [47].

We were able to assess physical health outcomes that are common in children and plausibly predicted by MHC, together with measured aspects of cognitive development. The MCS has data on many explanatory covariates which could be examined in relation to MHC, and accounted for when investigating associations between MHC and the physical health and cognitive development of the child. As with many cohort studies, missing data was an issue. Weights were used in all analyses to account for sample design and attrition up to age 11. In addition, missing MHC items were automatically imputed during the LCA procedure. Although there were missing data due to non-response on other items, the maternal-report sample comprised 92% of the MCS children who took part in the 11 year sweep, and we used multiple imputation to address missing covariate and outcome data. We have shown that a relationship between MHC and child health and cognitive development largely persisted after adjustment for potential confounding by socio-demographic characteristics and maternal psychological distress. Nevertheless it remains possible that these cross-sectional associations are susceptible to residual confounding or reverse causality.

## Conclusions

Positive mental health is not simply the absence of mental ill health [2, 8]. Nevertheless, there is limited and sometimes conflicting research linking positive mental health with child health at a population level. Our finding that high MHC is associated with better outcomes in indicators of physical health and cognitive development at the end of primary school suggests that MHC may hold potential for public health and academic improvement. Future research should develop and test other measures of MHC and at other ages, use longitudinal data to investigate mechanisms by which associations between MHC and health and cognitive development change over time, and investigate whether MHC (which may be modifiable through intervention) may hold the potential to buffer against social disadvantage.

## Supplementary information

**Additional file 1.** Analyses with complete cases samples (Tables S1-S4); Mental Health Competence Latent Class Analyses (Tables S5-S7); additional analyses with multiple imputation samples (Tables S8 and S9).

## Data Availability

The datasets analysed during the current study are available from UK Data Service, University of Essex and University of Manchester (DOI: 10.5255/UKDA-SN-7464-4).

## Abbreviations

AIC: Akaike information criterion
BAS: British Ability Scales
BIC: Bayesian information criterion
BMI: Body Mass Index
CANTAB: Cambridge Neuropsychological Test Automated Battery
CI: confidence interval
IOTF: International Obesity Task Force
ISAAC: International Study of Asthma and Allergies in Childhood
LCA: Latent Class Analysis
LS: learning skills
MAR: Missing at random
MCS: Millennium Cohort Study
MHC: Mental health competence
MHD: Mental health difficulties
PS: Prosocial behaviours
RRR: Relative risk ratio
SDQ: Strengths and Difficulties Questionnaire
