# Supplementary material for: Associations between mental health competence and indicators of physical health and cognitive development in eleven year olds: findings from the UK Millennium Cohort Study
Source: BMC Public Health. 2019 Nov 6;19:1461. doi: 10.1186/s12889-019-7789-7 (PMC6836461; doi:10.1186/s12889-019-7789-7)
Supplement: Supplementary file 1 — Additional file 1. Analyses with complete cases samples (Tables S1-S4); Mental Health Competence Latent Class Analyses (Tables S5-S7); additional analyses with multiple imputation samples (Tables S8 and S9). [file 12889_2019_7789_MOESM1_ESM.docx]

**SUPPLEMENTARY MATERIAL: ANALYSES WITH COMPLETE CASES SAMPLES**

| **Table S1. Characteristics of MHC classes in the UK Millennium Cohort Study, weighted row % (n): complete case samples** | | | | | | | | | | | |
| --- | --- | --- | --- | --- | --- | --- | --- | --- | --- | --- | --- |
|  | | Maternal-report | | | | | Teacher-report | | | | |
|  | | All children  (Column %) | High MHC (Hi PS; Hi LS) | High-Moderate MHC (Hi PS; Mod LS) | Moderate MHC (Mod PS; Mod LS) | Low MHC (Mod PS; Low LS) | All children  (Column %) | High MHC (Hi PS; Hi LS) | High-Moderate MHC (Hi PS; Mod LS) | Moderate MHC (Mod PS; Mod LS) | Low MHC (Mod PS; Low LS) |
| Gender | Male | 52 (6098) | 30 (1950) | 36 (2227) | 23 (1352) | 11 (569) | 51 (3381) | 26 (922) | 27 (907) | 23 (818) | 24 (734) |
|  | Female | 48 (5984) | 44 (2736) | 35 (2073) | 16 (897) | 5 (278) | 49 (3358) | 53 (1827) | 25 (818) | 15 (499) | 7 (214) |
| *Chi square test p-value* | | *-* | *<0.0001* | | | | *-* | *<0.0001* | | | |
| Ethnicity | White | 86 (10216) | 36 (3853) | 37 (3752) | 20 (1904) | 8 (707) | 87 (5611) | 39 (2304) | 27 (1470) | 19 (1066) | 15 (771) |
|  | Mixed | 3 (344) | 32 (128) | 36 (117) | 23 (70) | 9 (29) | 3 (229) | 38 (91) | 20 (50) | 21 (48) | 21 (40) |
|  | Indian | 2 (272) | 45 (122) | 30 (83) | 19 (52) | 6 (15) | 2 (188) | 44 (88) | 23 (46) | 19 (38) | 14 (16) |
|  | Pakistani/Bangladeshi | 4 (732) | 46 (325) | 28 (199) | 19 (145) | 8 (63) | 3 (386) | 37 (152) | 22 (81) | 25 (91) | 17 (62) |
|  | Black/ Black British | 3 (363) | 47 (176) | 30 (110) | 14 (53) | 9 (24) | 3 (229) | 32 (70) | 22 (62) | 22 (49) | 24 (48) |
|  | Other Ethnic group | 1 (152) | 48 (81) | 28 (39) | 18 (25) | 6 (7) | 1 (94) | 43 (44) | 19 (16) | 26 (25) | 12 (9) |
| *Chi square test p-value* | | *-* | *<0.0001* | | | | *-* | *0.05* | | | |
| Maternal age at MCS birth (years) | 14-19 | 9 (820) | 27 (218) | 41 (332) | 20 (168) | 13 (102) | 8 (397) | 26 (110) | 25 (96) | 23 (97) | 26 (94) |
|  | 20-29 | 45 (5275) | 34 (1896) | 36 (1927) | 20 (991) | 10 (461) | 44 (2880) | 37 (1099) | 27 (783) | 19 (552) | 17 (339) |
|  | 30-39 | 40 (5332) | 42 (2304) | 35 (1834) | 19 (958) | 5 (236) | 42 (3031) | 44 (1378) | 25 (730) | 20 (584) | 11 (339) |
|  | 40+ | 6 (655) | 38 (268) | 32 (207) | 21 (132) | 9 (48) | 6 (431) | 36 (162) | 29 (116) | 17 (84) | 18 (69) |
| *Chi square test p-value* | | *-* | *<0.0001* | | | | *-* | *<0.0001* | | | |
| Maternal academic qualification | Degree+ | 21 (2812) | 49 (1378) | 29 (838) | 18 (487) | 4 (109) | 23 (1619) | 53 (842) | 20 (329) | 19 (334) | 8 (114) |
|  | Diploma | 13 (1535) | 39 (590) | 36 (582) | 20 (289) | 5 (74) | 14 (913) | 45 (399) | 24 (225) | 19 (172) | 12 (117) |
|  | A-levels | 9 (1066) | 38 (421) | 38 (384) | 18 (202) | 7 (59) | 8 (529) | 47 (249) | 22 (117) | 17 (96) | 14 (67) |
|  | GCSE grade A*-C | 33 (3564) | 33 (1232) | 40 (1395) | 19 (673) | 8 (264) | 33 (2012) | 36 (763) | 30 (576) | 19 (382) | 15 (291) |
|  | GCSE D-G | 12 (1117) | 29 (354) | 37 (425) | 21 (208) | 13 (130) | 12 (678) | 29 (209) | 28 (193) | 22 (146) | 21 (130) |
|  | Other (including overseas) | 7 (742) | 35 (276) | 34 (245) | 20 (152) | 11 (69) | 7 (405) | 29 (132) | 22 (90) | 22 (88) | 27 (95) |
|  | None | 12 (1212) | 33 (423) | 34 (424) | 19 (229) | 13 (136) | 10 (566) | 25 (151) | 33 (191) | 16 (93) | 26 (131) |
| *Chi square test p-value* | | *-* | *<0.0001* | | | | *-* | *<0.0001* | | | |
| Family structure | Natural couple | 62 (8095) | 42 (3486) | 35 (2752) | 18 (1437) | 5 (420) | 63 (4534) | 44 (2049) | 25 (1116) | 19 (869) | 12 (500) |
|  | Reconstituted | 12 (1201) | 25 (305) | 40 (487) | 21 (260) | 14 (149) | 12 (695) | 32 (2277) | 28 (1315) | 19 (1005) | 22 (632) |
|  | Lone | 26 (2786) | 30 (895) | 37 (1061) | 21 (552) | 12 (278) | 25 (1510) | 29 (472) | 26 (410) | 21 (312) | 23 (316) |
| *Chi square test p-value* | | - | *<0.0001* | | | | *-* | *<0.0001* | | | |
| Maternal mental health | No-low distress | 57 (6674) | 43 (2978) | 35 (2287) | 18 (1138) | 5 (271) | 57 (3715) | 43 (1661) | 25 (919) | 19 (707) | 13 (428) |
|  | Med/high distress | 43 (4773) | 29 (1473) | 38 (1814) | 21 (978) | 12 (508) | 43 (2696) | 35 (983) | 26 (711) | 20 (545) | 19 (457) |
| *Chi square test p-value* | | *-* | *<0.0001* | | | | *-* | *<0.0001* | | | |
| **All children** | | **100 (12082)** | **37 (4686)** | **36 (4300)** | **19 (2249)** | **8 (847)** | **100 (6739)** | **39 (2749)** | **26 (1725)** | **19 (1317)** | **16 (948)** |

MHC: mental health competence; Hi PS: high prosocial behaviour; Mod PS: moderate PS; Hi LS: high learning skills; Mod LS: moderate learning skills; Low LS: low learning skills

Missing data (n): mother response: ethnicity 3, maternal highest academic qualification 34, maternal mental health 635; teacher response: ethnicity 2, maternal highest academic qualification 17, maternal mental health 328.

| **Table S2. Associations between reported mental health competence and mental health difficulties at age 11 in the UK Millennium Cohort Study, weighted column % (n): complete case samples** | | | | | | | | | |
| --- | --- | --- | --- | --- | --- | --- | --- | --- | --- |
|  |  | Maternal-report | | | | Teacher-report | | | |
|  |  | High MHC (Hi PS; Hi LS) | High-Moderate MHC (Hi PS; Mod LS) | Moderate MHC (Mod PS; Mod LS) | Low MHC (Mod PS; Low LS) | High MHC (Hi PS; Hi LS) | High-Moderate MHC (Hi PS; Mod LS) | Moderate MHC (Mod PS; Mod LS) | Low MHC (Mod PS; Low LS) |
| Mental health difficulties | No | 79 (3695) | 68 (2924) | 65 (1474) | 38 (333) | 76 (2097) | 58 (1025) | 66 (881) | 43 (419) |
|  | Yes | 21 (985) | 33 (1373) | 35 (768) | 62 (509) | 24 (649) | 42 (700) | 34 (435) | 57 (526) |

MHC: mental health competence; Hi PS: high prosocial behaviour; Mod PS: moderate PS; Hi LS: high learning skills; Mod LS: moderate learning skills; Low LS: low learning skills

Missing data (n): mother response: emotional problems 21; teacher response: emotional problems 7

| **Table S3. Associations between reported mental health competence and indicators of physical health and cognitive development at age 11 in the UK Millennium Cohort Study: complete case samples** | | | | | | | | | | | | |
| --- | --- | --- | --- | --- | --- | --- | --- | --- | --- | --- | --- | --- |
|  |  | Maternal-report | | | | Teacher-report | | | | | | |
|  |  | High MHC (Hi PS; Hi LS) | High-Moderate MHC (Hi PS; Mod LS) | Moderate MHC (Mod PS; Mod LS) | Low MHC (Mod PS; Low LS) | High MHC (Hi PS; Hi LS) | | High-Moderate MHC (Hi PS; Mod LS) | | Moderate MHC (Mod PS; Mod LS) | | Low MHC (Mod PS; Low LS) |
| **Weighted column % (n)** | | | | | | | | | | | | |
| Overweight | Healthy weight | 72 (3002) | 69 (2727) | 75 (1480) | 69 (501) | 73 (1809) | | 69 (1084) | | 71 (835) | | 70 (599) |
|  | Overweight | 22 (935) | 23 (919) | 19 (406) | 24 (173) | 22 (526) | | 24 (370) | | 22 (271) | | 21 (182) |
|  | Obese | 6 (272) | 8 (291) | 6 (131) | 7 (56) | 5 (125) | | 7 (119) | | 7 (92) | | 9 (77) |
| *Chi square test p-value* | *0.01* | | | | | *0.02* | | | | | | |
| Injury | None | 65 (3101) | 58 (2565) | 62 (1413) | 57 (505) | 64 (1787) | | 60 (1059) | | 63 (832) | | 55 (533) |
|  | 1 | 26 (1147) | 28 (1163) | 26 (579) | 27 (223) | 26 (702) | | 28 (465) | | 26 (329) | | 27 (261) |
|  | 2+ | 9 (423) | 14 (563) | 12 (251) | 16 (116) | 10 (259) | | 12 (200) | | 11 (155) | | 18 (154) |
| *Chi square test p-value* |  | *<0.0001* | | | | *<0.0001* | | | | | | |
| Asthma symptoms | None | 95 (4461) | 95 (4076) | 96 (2139) | 94 (793) | 95 (2623) | 94 (1636) | | | 96 (1263) | 95 (892) | |
|  | 1 | 3 (155) | 4 (151) | 3 (76) | 4 (31) | 4 (92) | 4 (64) | | | 3 (42) | 3 (31) | |
|  | 2+ | 1 (57) | 1 (65) | 1 (31) | 3 (21) | 1 (34) | 1 (25) | | | 1 (11) | 3 (25) | |
| *Chi square test p-value* | >0.05 | | | | | <0.05 | | | | | | |
| BAS verbal ability: Tertiles | High verbal skills | 34 (1703) | 27 (1193) | 27 (636) | 16 (133) | 35 (1001) | | | 26 (463) | 30 (406) | 18 (187) | |
|  | Medium verbal skills | 36 (1581) | 33 (1358) | 33 (735) | 30 (225) | 39 (1015) | | | 34 (540) | 34 (431) | 29 (258) | |
|  | Low verbal skills | 31 (1352) | 41 (1674) | 39 (822) | 54 (430) | 26 (717) | | | 41 (690) | 37 (466) | 53 (480) | |
| *Chi square test p-value* |  | *<0.0001* | | | | *<0.0001* | | | | | | |
| CANTAB Spatial working memory (strategy): Tertiles | Good strategy | 40 (1754) | 32 (1315) | 37 (766) | 25 (188) | 44 (1189) | | | 30 (506) | 39 (480) | 25 (230) | |
|  | Medium strategy | 35 (1578) | 35 (1477) | 35 (755) | 36 (269) | 33 (878) | | | 37 (612) | 34 (453) | 37 (232) | |
|  | Poor strategy | 25 (1142) | 32 (1316) | 29 (608) | 40 (309) | 23 (595) | | | 33 (551) | 27 (334) | 38 (337) | |
|  | *<0.0001* | | | | | *<0.0001* | | | | | | |
| CANTAB risk-taking: Tertiles | Low risk-taking | 37 (1701) | 32 (1370) | 31 (648) | 24 (180) | 40 (1063) | | | 29 (504) | 31 (387) | 24 (213) | |
|  | Medium risk-taking | 36 (1551) | 33 (1334) | 33 (709) | 33 (241) | 35 (919) | | | 36 (575) | 35 (431) | 30 (272) | |
|  | High risk-taking | 28 (1201) | 35 (1391) | 37 (764) | 43 (330) | 26 (669) | | | 35 (580) | 35 (442) | 46 (403) | |
| *Chi square test p-value* |  | *<0.0001* | | | | *<0.0001* | | | | | | |

MHC: mental health competence; Hi PS: high prosocial behaviour; Mod PS: moderate PS; Hi LS: high learning skills; Mod LS: moderate learning skills; Low LS: low learning skills

Missing data (n): mother response: overweight 394, injury 33, asthma 26, verbal ability 240, spatial working memory 605, risk taking scores 662; teacher response: overweight 176, injury 3, asthma 1, verbal ability 85, spatial working memory 251, risk taking scores 281.

| **Table S4. Associations between reported mental health competence and indicators of physical health and cognitive development at age 11 in the UK Millennium Cohort Study: complete case samples** | | | | | | | | | | |
| --- | --- | --- | --- | --- | --- | --- | --- | --- | --- | --- |
|  |  | Maternal-report | | | | Teacher-report | | | | |
|  |  | High MHC (Hi PS; Hi LS) | High-Moderate MHC (Hi PS; Mod LS) | Moderate MHC (Mod PS; Mod LS) | Low MHC (Mod PS; Low LS) | High MHC (Hi PS; Hi LS) | High-Moderate MHC (Hi PS; Mod LS) | Moderate MHC (Mod PS; Mod LS) | Low MHC (Mod PS; Low LS) | |
| **Unadjusted regression results: Relative risk ratios (95% CI)** | | | | | | | | | | |
| Overweight | Healthy weight | - | - | - | - | - | - | - | - | |
|  | Overweight | - | 1.0 (0.9-1.2) | 0.8 (0.7-1.0) | 1.2 (0.9-1.5) | - | 1.1 (0.9-1.4) | 1.1 (0.9-1.3) | 1.0 (0.8-1.3) | |
|  | Obese | - | 1.3 (1.1-1.6) | 0.9 (0.7-1.2) | 1.3 (0.9-1.9) | - | 1.4 (1.0-2.0) | 1.5 (1.1-2.1) | 1.8 (1.3-2.5) | |
| Injury | None | - | - | - | - |  | - | - | - | |
|  | 1 | - | 1.2 (1.1-1.4) | 1.1 (0.9-1.2) | 1.2 (1.0-1.5) | - | 1.2 (1.0-1.4) | 1.0 (0.9-1.2) | 1.2 (1.0-1.5) | |
|  | 2+ | - | 1.7 (1.5-2.0) | 1.5 (1.2-1.9) | 1.8 (1.3-2.6) | - | 1.3 (1.0-1.7) | 1.2 (0.9-1.5) | 2.3 (1.7-3.2) | |
| Asthma symptoms | None | **-** | **-** | **-** | **-** | **-** | **-** | **-** | | **-** |
|  | 1 | **-** | 1.1 (0.8-1.5) | 1.0 (0.7-1.3) | 1.2 (0.7-1.9) | - | 1.2 (0.8-1.6) | 0.8 (0.5-1.3) | 0.8 (0.5-1.4) | |
|  | 2+ | **-** | 1.0 (0.6-1.5) | 0.8 (0.5-1.4) | 1.9 (1.0-3.9) | - | 1.2 (0.7-2.2) | 0.7 (0.3-1.6) | 2.4 (1.2-4.7) | |
| BAS Verbal ability: tertiles | High verbal skills | - | - | - | - | - | - | - | - | |
|  | Medium verbal skills | - | 1.2 (1.0-1.3) | 1.2 (1.0-1.4) | 1.9 (1.5-2.5) |  | 1.1 (0.9-1.4) | 1.0 (0.8-1.2) | 1.4 (1.1-1.8) | |
|  | Low verbal skills | - | 1.8 (1.5-2.0) | 1.7 (1.4-2.0) | 4.0 (3.1-5.2) | - | 2.0 (1.6-2.4) | 1.5 (1.3-1.9) | 3.7 (2.9-4.7) | |
| CANTAB Spatial working memory (strategy): tertiles | Good strategy | - | - | - | - | - | - | - | - | |
|  | Medium strategy | - | 1.2 (1.1-1.4) | 1.1 (0.9-1.2) | 1.6 (1.2-2.0) | - | 1.6 (1.4-1.9) | 1.1 (1.0-1.4) | 1.9 (1.5-2.5) | |
|  | Poor strategy | - | 1.6 (1.4-1.9) | 1.3 (1.1-1.5) | 2.7 (2.1-3.5) | - | 2.1 (1.7-2.6) | 1.4 (1.1-1.7) | 2.9 (2.3-3.7) | |
| CANTAB risk-taking scores: tertiles | Low risk-taking | - | - | - | - | - | - | - | - | |
|  | Medium risk-taking | - | 1.1 (1.0-1.3) | 1.1 (0.9-1.4) | 1.5 (1.1-2.0) | - | 1.5 (1.2-1.8) | 1.3 (1.1-1.6) | 1.5 (1.1-1.9) | |
|  | High risk-taking | - | 1.5 (1.3-1.7) | 1.6 (1.4-1.9) | 2.6 (1.9-3.4) | - | 2.0 (1.7-2.4) | 1.8 (1.4-2.2) | 3.1 (2.4-4.1) | |
| **Adjusted regression results*: Relative risk ratios (95% CI)** | | | | | | | | | | |
| Overweight | Healthy weight | - | - | - | - | - | - | - | - | |
|  | Overweight | - | 1.0 (0.9-1.2) | 0.8 (0.7-1.0) | 1.1 (0.9-1.5) | - | 1.1 (0.9-1.4) | 1.1 (0.9-1.4) | 1.0 (0.8-1.4) | |
|  | Obese | - | 1.2 (1.0-1.5) | 0.9 (0.7-1.1) | 1.0 (0.7-1.5) | - | 1.3 (0.9-1.8) | 1.4 (1.0-2.0) | 1.5 (1.0-2.2) | |
| Injury | None | - | - | - | - |  | - | - | - | |
|  | 1 | - | 1.2 (1.0-1.3) | 1.0 (0.9-1.2) | 1.1 (0.9-1.4) | - | 1.2 (1.0-1.4) | 1.0 (0.9-1.2) | 1.2 (1.0-1.4) | |
|  | 2+ | - | 1.6 (1.3-1.8) | 1.4 (1.1-1.7) | 1.5 (1.1-2.2) | - | 1.3 (1.0-1.6) | 1.1 (0.9-1.5) | 2.2 (1.6-3.0) | |
| Asthma symptoms | None | - | - | - | - | - | - | - | - | |
|  | 1 | - | 1.0 (0.8-1.4) | 0.9 (0.6-1.2) | 1.0 (0.6-1.6) | - | 1.1 (0.8-1.6) | 0.7 (0.5-1.2) | 0.7 (0.4-1.3) | |
|  | 2+ | - | 0.8 (0.5-1.3) | 0.7 (0.4-1.2) | 1.4 (0.7-2.8) | - | 1.0 (0.6-1.7) | 0.5 (0.2-1.2) | 1.5 (0.7-3.1) | |
| BAS Verbal ability: tertiles | High verbal skills | - | - | - | - | - | - | - | - | |
|  | Medium verbal skills | - | 1.1 (0.9-1.2) | 1.1 (0.9-1.3) | 1.6 (1.2-2.2) |  | 1.1 (0.9-1.3) | 1.0 (0.8-1.2) | 1.3 (1.0-1.7) | |
|  | Low verbal skills | - | 1.5 (1.4-1.8) | 1.6 (1.3-1.8) | 2.8 (2.1-3.6) | - | 1.7 (1.4-2.1) | 1.5 (1.2-1.9) | 3.0 (2.4-3.9) | |
| CANTAB Spatial working memory (strategy): tertiles | Good strategy | - | - | - | - | - | - | - | - | |
|  | Medium strategy | - | 1.2 (1.0-1.3) | 1.0 (0.9-1.2) | 1.4 (1.1-1.8) | - | 1.6 (1.3-1.9) | 1.1 (1.0-1.4) | 1.8 (1.4-2.3) | |
|  | Poor strategy | - | 1.5 (1.3-1.8) | 1.2 (1.0-1.5) | 2.2 (1.7-2.8) | - | 2.0 (1.6-2.4) | 1.3 (1.0-1.6) | 2.5 (2.0-3.3) | |
| CANTAB risk taking: tertiles | Low risk-taking | - | - | - | - | - | - | - | - | |
|  | Medium risk-taking | - | 1.0 (0.9-1.2) | 1.0 (0.8-1.2) | 1.2 (0.9-1.7) | - | 1.3 (1.1-1.6) | 1.1 (0.9-1.3) | 1.1 (0.9-1.5) | |
|  | High risk-taking | - | 1.3 (1.2-1.5) | 1.3 (1.1-1.5) | 1.9 (1.4-2.5) | - | 1.6 (1.4-1.9) | 1.2 (1.0-1.5) | 1.9 (1.4-2.4) | |

MHC: mental health competence; Hi PS: high prosocial behaviour; Mod PS: moderate PS; Hi LS: high learning skills; Mod LS: moderate learning skills; Low LS: low learning skills

*Adjusted for cohort member gender and ethnicity, maternal age at birth of cohort child, maternal academic attainment, maternal mental health and family structure

Missing data (n): mother response: ethnicity 3, maternal highest academic qualification 34, maternal mental health 635, overweight 394, injury 33, asthma 26, verbal ability 240, spatial working memory 605, risk taking scores 662; teacher response: ethnicity 2, maternal highest academic qualification 17, maternal mental health 328, overweight 176, injury 3, asthma 1, verbal ability 85, spatial working memory 251, risk taking scores 281.

**SUPPLEMENTARY MATERIAL: MENTAL HEALTH COMPETENCE LATENT CLASS ANALYSES**

MHC items fall broadly into the domains of learning skills and prosocial behaviours (listed in Table S5).

| **Table S5. Items relating to mental health competence in the UK Millennium Cohort Study using maternal and teacher reports** | | |
| --- | --- | --- |
| *MHC domain* | *Maternal report* | *Teacher*  *report* |
|  |  |  |
| *Prosocial behaviour* |  |  |
| SDQ Prosocial subscale items:   - Child is considerate of other people's feelings (“Considerate”: not true, somewhat true, certainly true) - Child shares readily with other children (treats, toys, pencils etc.) (“Shares”: not true, somewhat true, certainly true) - Child is helpful if someone is hurt, upset or feeling ill (“Helpful”: not true, somewhat true, certainly true) - Child is kind to younger children (“Kind”: not true, somewhat true, certainly true) - Child often volunteers to help others (parents, teachers, other children) (“Volunteers”: not true, somewhat true, certainly true) | X  X  X  X  X | X  X  X  X  X |
| *Learning skills* |  |  |
| SDQ Conduct problems subscale items:   - Child is generally obedient, usually does what adults request (“Obedient”: not true, somewhat true, very true)   SDQ Hyperactivity subscale items:   - Child sees tasks through to the end (“Tasks”: not true, somewhat true, certainly true) - Child thinks things out before acting (“Thinks”: not true, somewhat true, certainly true)   Other items:   - Child works well independently (“Independent” : not well at all, not very well, very well) - Child tries their best at school (“Tries best”: never, sometimes, always) | X  X  X | X  X  X  X  X |

A number of factors were used to select the optimal number of classes for the mother and teacher reported measures of mental health competence, including the following measures of model fit: Akaike information criterion (AIC), Bayesian information criterion (BIC), class posterior probabilities (likelihood of members of an assigned class belonging to that class), and entropy (the precision of membership assignment across all individuals).

These are shown for two to seven class measures in Tables S6 (mother) and S7 (teacher). The selection of the final four class MHC measure was based on both model fit and interpretability of the classes.

| **Table S6. Factors used to assess the latent class models: maternal-report** | | | | | | | |
| --- | --- | --- | --- | --- | --- | --- | --- |
|  | **1 class** | **2 class** | **3 class** | **4 class** | **5 class** | **6 class** | **7 class** |
| *Posterior probabilities*:* | | | | | | | |
|  | 1.00 | 0.90 | 0.87 | 0.78 | 0.78 | 0.67 | 0.68 |
|  |  | 0.94 | 0.81 | 0.79 | 0.77 | 0.73 | 0.71 |
|  |  |  | 0.88 | 0.88 | 0.84 | 0.81 | 0.77 |
|  |  |  |  | 0.84 | 0.86 | 0.85 | 0.85 |
|  |  |  |  |  | 0.72 | 0.76 | 0.54 |
|  |  |  |  |  |  | 0.76 | 0.69 |
|  |  |  |  |  |  |  | 0.78 |
| *Entropy^:* | | | | | | | |
|  | 1 | 0.76 | 0.67  67 | 0.68 | 0.68 | 0.66 | 0.63 |
| *BIC^^:* | | | | | | | |
|  |  | 7611 | 5611 | 4797 | 4478 | 4350 | 4380 |
| *AIC^^:* | | | | | | | |
|  |  | 7366 | 5241 | 4300 | 3857 | 3603 | 3507 |

*1 indicates perfect assignment within that class; ^1 indicates perfect assignment of all individuals to all classes; *^^*lower values indicate a more parsimonious model

| **Table S7. Factors used to assess the latent class models: teacher-report** | | | | | | | |
| --- | --- | --- | --- | --- | --- | --- | --- |
|  | **1 class** | **2 class** | **3 class** | **4 class** | **5 class** | **6 class** | **7 class** |
| *Posterior probabilities*:* | | | | | | | |
|  | 1.00 | 0.97 | 0.91 | 0.87 | 0.84 | 0.83 | 0.83 |
|  |  | 0.96 | 0.94 | 0.88 | 0.92 | 0.89 | 0.88 |
|  |  |  | 0.94 | 0.95 | 0.88 | 0.87 | 0.83 |
|  |  |  |  | 0.93 | 0.85 | 0.86 | 0.82 |
|  |  |  |  |  | 0.94 | 0.93 | 0.93 |
|  |  |  |  |  |  | 0.82 | 0.83 |
|  |  |  |  |  |  |  | 0.85 |
| *Entropy^:* | | | | | | | |
|  | 1 | 0.88 | 0.84 | 0.84 | 0.83 | 0.83 | 0.82 |
| *BIC^^:* | | | | | | | |
|  |  | 16342 | 12360 | 10357 | 9403 | 8959 | 8971 |
| *AIC^^:* | | | | | | | |
|  |  | 16036 | 11896 | 9737 | 8626 | 8026 | 7877 |

*1 indicates perfect assignment within that class; ^1 indicates perfect assignment of all individuals to all classes; *^^*lower values indicate a more parsimonious model

**SUPPLEMENTARY MATERIAL: ADDITIONAL ANALYSES WITH MULTIPLE IMPUTATION SAMPLES**

| **Table S8. Associations between reported mental health competence and indicators of physical health and cognitive development at age 11 in the UK Millennium Cohort Study: multiple imputation samples** | | | | | | | | | | | | |
| --- | --- | --- | --- | --- | --- | --- | --- | --- | --- | --- | --- | --- |
|  |  | Maternal-report (n=12082) | | | | Teacher-report (n=6739) | | | | | | |
|  |  | High MHC (Hi PS; Hi LS) | High-Moderate MHC (Hi PS; Mod LS) | Moderate MHC (Mod PS; Mod LS) | Low MHC (Mod PS; Low LS) | High MHC (Hi PS; Hi LS) | | High-Moderate MHC (Hi PS; Mod LS) | | Moderate MHC (Mod PS; Mod LS) | | Low MHC (Mod PS; Low LS) |
| **Weighted column %** | | | | | | | | | | | | |
| Overweight | Healthy weight | 72 | 69 | 75 | 69 | 73 | | 69 | | 70 | | 70 |
|  | Overweight | 22 | 23 | 19 | 24 | 22 | | 24 | | 22 | | 21 |
|  | Obese | 6 | 8 | 6 | 8 | 5 | | 7 | | 8 | | 9 |
| *Chi square test p-value* | *<0.001* | | | | | *<0.01* | | | | | | |
| Injury | None | 65 | 59 | 62 | 59 | 64 | | 60 | | 63 | | 56 |
|  | 1 | 26 | 29 | 26 | 28 | 27 | | 29 | | 26 | | 27 |
|  | 2+ | 8 | 13 | 12 | 13 | 9 | | 11 | | 10 | | 17 |
| *Chi square test p-value* |  | *<0.0001* | | | | *<0.0001* | | | | | | |
| Asthma symptoms | None | 95 | 95 | 96 | 94 | 95 | 94 | | | 96 | 95 | |
|  | 1 | 3 | 4 | 3 | 4 | 3 | 4 | | | 3 | 3 | |
|  | 2+ | 1 | 1 | 1 | 3 | 1 | 1 | | | 1 | 3 | |
| *Chi square test p-value* | >0.05 | | | | | <0.05 | | | | | | |
| BAS verbal ability: Tertiles | High verbal skills | 40 | 32 | 32 | 22 | 42 | | | 32 | 36 | 23 | |
|  | Medium verbal skills | 37 | 37 | 37 | 34 | 39 | | | 37 | 35 | 35 | |
|  | Low verbal skills | 23 | 31 | 31 | 44 | 19 | | | 32 | 29 | 42 | |
| *Chi square test p-value* |  | *<0.0001* | | | | *<0.0001* | | | | | | |
| CANTAB Spatial working memory (strategy): Tertiles | Good strategy | 33 | 26 | 30 | 18 | 37 | | | 22 | 32 | 20 | |
|  | Medium strategy | 34 | 34 | 33 | 33 | 33 | | | 36 | 32 | 32 | |
|  | Poor strategy | 34 | 41 | 38 | 49 | 30 | | | 42 | 35 | 48 | |
|  | *<0.0001* | | | | | *<0.0001* | | | | | | |
| CANTAB risk-taking: Tertiles | Low risk-taking | 34 | 30 | 29 | 23 | 38 | | | 27 | 29 | 23 | |
|  | Medium risk-taking | 35 | 33 | 32 | 31 | 35 | | | 35 | 34 | 29 | |
|  | High risk-taking | 30 | 37 | 39 | 46 | 27 | | | 38 | 37 | 47 | |

MHC: mental health competence; Hi PS: high prosocial behaviour; Mod PS: moderate PS; Hi LS: high learning skills; Mod LS: moderate learning skills; Low LS: low learning skills

| *Chi square test p-value* |  | | *<0.0001* | | | | *<0.0001* | | | | |
| --- | --- | --- | --- | --- | --- | --- | --- | --- | --- | --- | --- |
| **Table S9. Associations between reported mental health competence and indicators of physical health and cognitive development in the UK Millennium Cohort Study, adjusted models accounting for covariates including mental health difficulties: Multiple imputation samples** | | | | | | | | | | | |
|  |  | Maternal-report (n=12082) | | | | | Teacher-report (n=6739) | | | | |
|  |  | High MHC (Hi PS; Hi LS) | | High-Moderate MHC (Hi PS; Mod LS) | Moderate MHC (Mod PS; Mod LS) | Low MHC (Mod PS; Low LS) | High MHC (Hi PS; Hi LS) | High-Moderate MHC (Hi PS; Mod LS) | Moderate MHC (Mod PS; Mod LS) | Low MHC (Mod PS; Low LS) | |
| **Unadjusted Relative risk ratios (95% CI)** | | | | | | | | | | | |
| Overweight | Healthy weight | - | | - | - | - | - | - | - | - | |
|  | Overweight | - | | 1.1 (0.9-1.2) | 0.8 (0.7 -1.0) | 1.1 (0.9-1.4) | - | 1.2 (1.0-1.4) | 1.1 (0.9-1.3) | 1.0 (0.8-1.3) | |
|  | Obese | - | | 1.3 (1.1-1.6) | 1.0 (0.8-1.3) | 1.3 (0.9-1.9) | - | 1.5 (1.1-2.0) | 1.5 (1.1-2.1) | 1.7 (1.2-2.4) | |
| Injury | None | - | | - | - | - |  | - | - | - | |
|  | 1 | - | | 1.2 (1.1- 1.4) | 1.0 (0.9- 1.2) | 1.2 (1.0-1.4) | - | 1.2 (1.0-1.4) | 1.0 (0.9-1.2) | 1.2 (1.0-1.4) | |
|  | 2+ | - | | 1.7 (1.5-2.0) | 1.5 (1.2-1.8) | 1.8 (1.3-2.4) | - | 1.3 (1.0-1.6) | 1.2 (0.9-1.5) | 2.1 (1.6-2.9) | |
| Asthma symptoms | None | **-** | | **-** | **-** | **-** | **-** | **-** | **-** | | **-** |
|  | 1 | **-** | | 1.1 (0.9-1.5) | 1.0 (0.7-1.4) | 1.1 (0.7-1.8) | - | 1.2 (0.9-1.7) | 0.8 (0.5-1.3) | 0.8 (0.5-1.4) | |
|  | 2+ | **-** | | 1.0 (0.6-1.5) | 0.8 (0.5-1.4) | 2.0 (1.0-3.8) | - | 1.2 (0.7-2.1) | 0.7 (0.3-1.6) | 2.2 (1.2-4.3) | |
| BAS Verbal ability: tertiles | High verbal skills | - | | - | - | - | - | - | - | - | |
|  | Medium verbal skills | - | | 1.3 (1.2-1.5) | 1.3 (1.1-1.5) | 1.7 (1.4-2.2) |  | 1.3 (1.1-1.5) | 1.1 (0.9-1.3) | 1.6 (1.3-2.0) | |
|  | Low verbal skills | - | | 1.8 (1.5-2.0) | 1.7 (1.5-2.0) | 3.7 (2.8-4.7) | - | 2.3 (1.9-2.7) | 1.8 (1.5-2.2) | 4.0 (3.2-5.0) | |
| CANTAB Spatial working memory (strategy): tertiles | Good strategy | - | | - | - | - | - | - | - | - | |
|  | Medium strategy | - | | 1.3 (1.1-1.5) | 1.1 (0.9-1.3) | 1.7 (1.3-2.3) | - | 1.8 (1.5-2.1) | 1.1 (0.9-1.4) | 1.9 (1.5-2.4) | |
|  | Poor strategy | - | | 1.5 (1.3-1.8) | 1.2 (1.0-1.5) | 2.6 (2.1-3.3) | - | 2.3 (1.9-2.8) | 1.4 (1.1-1.6) | 3.0 (2.5-3.7) | |
| CANTAB risk taking scores: tertiles | Low risk-taking | - | | - | - | - | - | - | - | - | |
|  | Medium risk-taking | - | | 1.1 (0.9-1.2) | 1.1 (0.9-1.3) | 1.3 (1.0-1.7) | - | 1.4 (1.2-1.7) | 1.3 (1.1-1.6) | 1.4 (1.1-1.7) | |
|  | High risk-taking | - | | 1.4 (1.2-1.6) | 1.5 (1.3-1.8) | 2.3 (1.8-3.0) | - | 2.0 (1.7-2.3) | 1.8 (1.5-2.2) | 2.8 (2.2-3.6) | |
| **Adjusted Relative risk ratios* (95% CI)** | | | | | | | | | | | |
| Overweight | Healthy weight | - | | - | - | - | - | - | - | - | |
|  | Overweight | - | | 1.1 (0.9-1.2) | 0.8 (0.7-1.0) | 1.1 (0.8-1.4) | - | 1.2 (1.0-1.4) | 1.1 (0.9-1.4) | 1.0 (0.8-1.4) | |
|  | Obese | - | | 1.2 (1.0-1.4) | 0.9 (0.7-1.1) | 0.9 (0.6-1.3) | - | 1.2 (0.9-1.7) | 1.4 (1.0-2.0) | 1.3 (0.9-1.9) | |
| Injury | None | - | | - | - | - |  | - | - | - | |
|  | 1 | - | | 1.2 (1.0-1.3) | 1.0 (0.9-1.1) | 1.1 (0.9-1.3) | - | 1.2 (1.0-1.4) | 1.0 (0.9-1.2) | 1.2 (1.0-1.4) | |
|  | 2+ | - | | 1.5 (1.3-1.8) | 1.3 (1.0-1.6) | 1.4 (1.0-1.9) | - | 1.2 (1.0-1.6) | 1.1 (0.9-1.5) | 2.0 (1.5-2.8) | |
| Asthma symptoms | None | - | | - | - | - | - | - | - | - | |
|  | 1 | - | | 1.0 (0.8-1.3) | 0.9 (0.6-1.2) | 0.8 (0.5-1.2) | - | 1.1 (0.8-1.6) | 0.7 (0.5-1.2) | 0.7 (0.4-1.2) | |
|  | 2+ | - | | 0.8 (0.5-1.2) | 0.6 (0.4-1.1) | 1.1 (0.6-2.3) | - | 0.9 (0.5-1.5) | 0.5 (0.2-1.2) | 1.1 (0.5-2.4) | |
| BAS Verbal ability: tertiles | High verbal skills | - | | - | - | - | - | - | - | - | |
|  | Medium verbal skills | - | | 1.2 (1.1-1.3) | 1.2 (1.0-1.4) | 1.4 (1.1-1.8) |  | 1.2 (1.0-1.4) | 1.1 (0.9-1.3) | 1.4 (1.1-1.8) | |
|  | Low verbal skills | - | | 1.5 (1.3-1.7) | 1.5 (1.3-1.8) | 2.2 (1.7-2.9) | - | 1.9 (1.6-2.2) | 1.7 (1.4-2.2) | 3.0 (2.3-3.8) | |
| CANTAB Spatial working memory (strategy): tertiles | Good strategy | - | | - | - | - | - | - | - | - | |
|  | Medium strategy | - | | 1.2 (1.1-1.4) | 1.0 (0.9-1.2) | 1.5 (1.1-1.9) | - | 1.7 (1.4-2.0) | 1.1 (0.9-1.3) | 1.6 (1.2-2.1) | |
|  | Poor strategy | - | | 1.4 (1.2-1.6) | 1.1 (0.9-1.3) | 1.9 (1.5-2.5) | - | 2.1 (1.7-2.5) | 1.3 (1.0-1.6) | 2.4 (1.9-3.1) | |
| CANTAB risk-taking: tertiles | Low risk-taking | - | | - | - | - | - | - | - | - | |
|  | Medium risk-taking | - | | 1.0 (0.9- 1.2) | 1.0 (0.8- 1.2) | 1.1 (0.8-1.5) | - | 1.3 (1.1-1.6) | 1.1 (0.9- 1.3) | 1.0 (0.8-1.4) | |
|  | High risk-taking | - | | 1.3 (1.1-1.4) | 1.2 (1.0-1.5) | 1.7 (1.2-2.2) | - | 1.6 (1.3-1.9) | 1.3 (1.0-1.6) | 1.7 (1.3-2.2) | |

MHC: mental health competence; Hi PS: high prosocial behaviour; Mod PS: moderate PS; Hi LS: high learning skills; Mod LS: moderate learning skills; Low LS: low learning skills

*Adjusted for cohort member gender and ethnicity, maternal age at birth of cohort child, maternal academic attainment, maternal mental health and family structure, mental health difficulties
